# Pushing the Boundaries of Pedicled Chest Wall Perforator Flaps in Oncoplastic Breast Surgery

**DOI:** 10.7759/cureus.36686

**Published:** 2023-03-26

**Authors:** Ishita Laroiya, Melissa Tan, Shaista Zafar, Geeta Shetty

**Affiliations:** 1 Breast Surgery, Birmingham City Hospital, Birmingham, GBR

**Keywords:** patient-reported outcomes, implant salvage procedures, upper inner quadrant tumours, central quadrant tumours, breast reconstruction, chest wall perforator flaps

## Abstract

Among 145 chest wall perforator flaps (CWPFs) performed at City Hospital, Birmingham (September 2017-February 2022), 11 were for novel indications, four were for whole breast reconstructions, two were for implant salvage procedures, three were CWPFs with skin paddle to replace excised skin/nipple-areola complex, and two were for upper inner quadrant tumours.

Tumour characteristics and post-operative complications were noted. Patient-reported outcomes measures (PROMs) were measured using a questionnaire adapted from the National Mastectomy and Breast Reconstruction Audit (NMBRA) study.

Among 11 patients, nine (81.81%) did not develop any complications. Ten patients responded to PROMs (median follow-up of eight months). The PROMs assessment showed that all patients (100%) were satisfied with the post-operative breast appearance. Of the patients, 90% (9/10) felt the results of their surgery to be good, very good, or excellent. Of the patients, 70% (7/10) said that they have no/little persistent pain. None of the patients had difficulty carrying out normal activities.

Thus, the applications of CWPFs could be extended for whole breast reconstruction, implant salvage procedures, where skin paddle is needed, and for upper inner quadrant tumours.

## Introduction

Some areas in breast surgery continue to be frontiers, which challenge our ability to deliver the best care to our patients. The current whole breast reconstruction techniques, be it implants or free flaps, come with their own set of complications [1]. Also, the upper inner quadrant (UIQ) continues to be an area of the breast that still challenges breast surgeons, especially in patients with small/moderate breasts [2]. Another problematic area is when skin excision is required if the tumour is close to the skin or nipple-areola complex (NAC). Traditionally, the latissimus dorsi (LD) flap with skin has been the workhorse flap for this [3]. However, LD flap has its own morbidity associated with muscle harvests like seroma, donor site wound problems, and some difficulty in exercise with the ipsilateral arm for at least some time [4,5]; hence, muscle-sparing options like chest wall perforator flaps (CWPFs) hold potential for use in these situations. Some authors have also described the use of free flaps like deep inferior epigastric perforator (DIEP) flaps, but these again come with donor site morbidity and prolonged operative times [6]. Implant salvage is another area where innovative options are needed, especially if skin cover is vital for an exposed implant.

Since the initial description by Hamdi et al. for partial breast reconstruction, CWPFs have become the modality of choice for the same [7]. CWPFs score over traditional choices like LD flap as donor site morbidity is reduced. We have adopted CWPF to provide solutions for the above problems, which is a novel concept.

## Materials and methods

From September 2017 to February 2022, 145 patients underwent CWPFs at the City Hospital, Birmingham, UK. Out of these, 11 patients underwent CWPFs for novel indications: whole breast reconstruction, implant salvage procedures, and flaps done for UIQ tumours and where skin paddle is required. These were included in the analysis.

All cancer patients were diagnosed and treated for breast cancer as per the standard protocol [8]. Using a pre-existing database, the characteristics of the patients, the tumour (where applicable), and post-operative complications (immediate and within 30 days) were noted. The complications were classified as per the Clavien-Dindo classification [9].

The patients were either interviewed (retrospectively accrual of patients) at clinical follow-up or interviewed telephonically, after verbal consent. Patients were required to fill out a patient-reported outcomes measures (PROMs) questionnaire, which comprehensively required them to grade their level of satisfaction with the services, with the surgeon, the team, their post-operative result, and any impact of the surgery on their physical, emotional, or sexual well-being. The questionnaire was adapted from the National Mastectomy and Breast Reconstruction Audit (NMBRA) study for use in CWPFs since no questionnaire exists for use specifically for CWPFs (Appendix) [10].

We have used a questionnaire already validated for use in the UK and PROMs assessment are now being used as a part of patient care as per National Health Service (NHS) recommendations [11]. Therefore, ethical approval was not needed for the study. The study complied with NHS Trust Governance and Audit Department guidelines. One patient could not be contacted. Hence, PROMs were available for 10 patients.

The primary aim of the study was to evaluate the PROMs. The secondary aim was to evaluate the surgical outcomes in this patient cohort.

Descriptive analysis (mean and percentages) were used to analyse the data.

Operative technique

Whole Breast Reconstruction

Patients with small breasts (A/B cup size) who had good tissue volume available at the lateral fold and in the upper abdomen (assessed by skin pinch test) were recruited for this procedure.

Nipple-sparing mastectomy was performed using the hydro-dissection technique [12]. The axillary procedure was performed as per standard guidelines [8]. After mastectomy, whole-breast reconstruction was performed using two CWPFs: lateral intercostal artery perforator flap (LICAP)/lateral thoracic artery perforator flap (LTAP) and anterior intercostal artery perforator flap (AICAP). The breast mound was then reconstructed by insetting both the flaps into the mastectomy defect.

Volume Replacement for UIQ Tumours

Wide local excision (WLE) of the tumour with margins was performed through a peri-areolar approach. After this, the resultant defect was filled with CWPFs based on the medial intercostal artery perforator flap (MICAP).

Skin Cover for Tumours Where Skin or Nipple-Areolar Complex Was Excised

For the patients where skin excision was required in the outer half of the breast, they underwent LTAP/LICAP to provide volume replacement and skin cover after tumour excision. LTAP/LICAP was harvested as per standard technique and was rotated in a propeller fashion into the defect [13].

For the patients where NAC excision was required along with the tumour, after standard WLE with NAC excision, AICAP/MICAP was harvested and was used to replace volume as well as replace the areolar skin.

Implant Salvage Procedures

Implant salvage procedures were carried out in two patients, who had implant extrusion on native skin. One of them had an infected implant, which was replaced with another after wash-out and a MICAP flap was used to provide skin cover. The second patient had already undergone multiple procedures for capsular contracture and implant extrusion. Hence, the implant was removed and replaced with a LICAP flap with a skin island.

## Results

**Figure 1:**
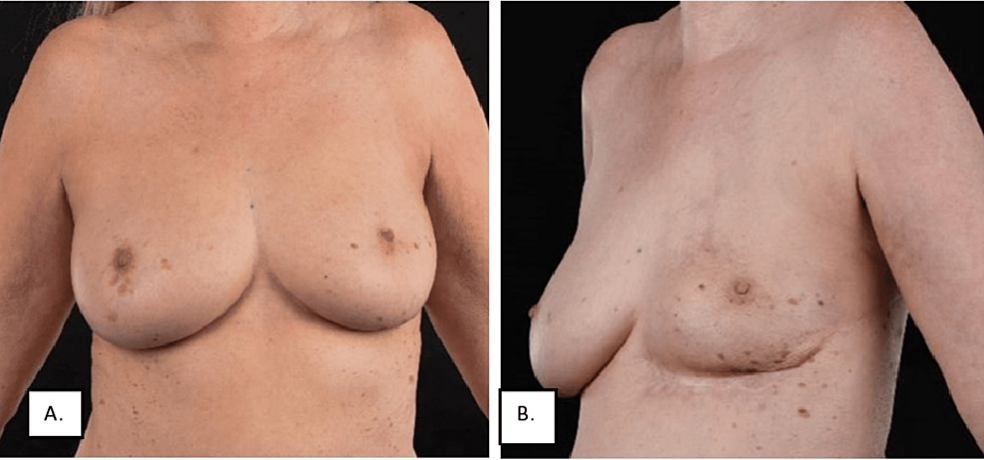
(A) Pre-operative photograph. (B) Eight months post-left NSM with whole breast reconstruction with LICAP + MICAP flaps NSM: nipple-sparing mastectomy; LICAP: lateral intercostal artery perforator flap; MICAP: medial intercostal artery perforator flap.

**Figure 2:**
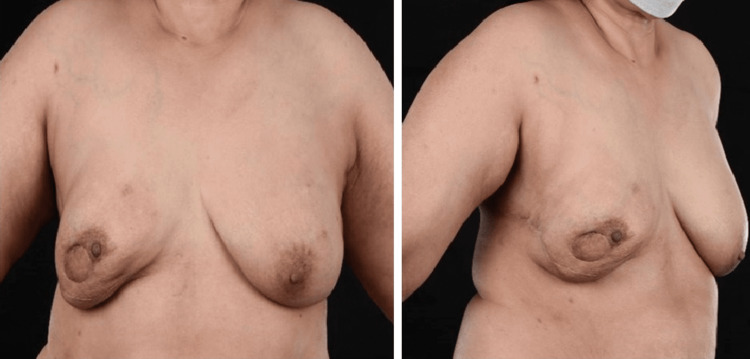
Post-operative photographs (three months post-operative) of patients who underwent LICAP (with skin) for implant salvage (implant put for augmentation was removed and replaced with LICAP) LICAP: lateral intercostal artery perforator flap.

**Figure 3:**
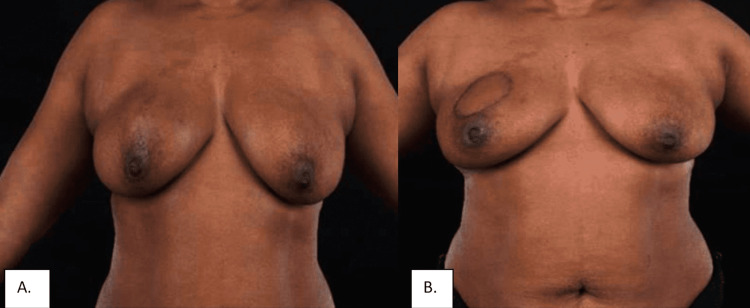
(A) Pre-operative photograph of the patient with large UOQ and central quadrant tumour close to the skin. (B) One month post-operative photo of the patient post-WLE + ANC + LTAP with skin UOQ: upper outer quadrant; WLE: wide local excision; ANC: axillary node clearance; LTAP: lateral thoracic artery perforator flap.

A total of 145 patients underwent CWPFs in the City Hospital, Birmingham, UK from September 2017 to February 2022. Out of these, 11 patients underwent CWPFs for novel indications, four patients for whole breast reconstruction (Figure 1), two patients for implant salvage procedures (Figure 2), two patients for volume replacement in UIQ, and three patients for providing skin cover for tumours where skin or NAC was excised (Figure 3).

The mean age of the patients was 51.73 years (±7.47 years). The mean pathological invasive tumour size was 43.85 mm (±31.62). The detailed clinicopathological features of the patients are discussed in Table 1.

**Table 1:** Clinicopathological characteristics of the patients NAC: nipple-areola complex; UIQ: upper inner quadrant; DCIS: ductal carcinoma in situ; IDC: Infiltrating Ductal Carcinoma; ILC: infiltrating lobular carcinoma.

Whole breast reconstruction					
	Age (years)	Stage	Histology	Pathological tumour size (mm)	Pathological nodal status
1	55	IA	Invasive micropapillary tumour (with DCIS)	2.5 - invasive; whole tumour = 110	0/2 (N0)
2	57	IIA	IDC	20	0/1 (N0)
3	63	IA	IDC	9	0/2 (N0)
4	53	0	DCIS - high grade (HG)	78	0/1 (N0)
Salvage procedure for implant complications					
1	55	NA	NA	NA	NA
2	56	Recurrence	ILC	25	NA
Providing skin cover (for skin/NAC)					
1	44	IIB	IDC	80	0/3 (N0)
2	55	IIA	IDC (with DCIS)	15; whole tumour = 75	0/2 (N0)
3	46	IIA	IDC (with HG DCIS)	50	0/1 (N0)
UIQ tumours					
1	34	IIIA	IDC	2.5; 40 (multi-focal; whole area = 68)	5/11 (N2)
2	51	0	HG DCIS	91	NA

The median follow-up was eight months (range = 2-17 months). None of these patients had a recurrence or distant metastasis during this period.

Of the patients, 9/11 (81.81%) underwent surgery without any complications. Of the patients, 2/11 (18.18%) had complications: one patient with whole breast reconstruction had skin flap necrosis and hence, delayed wound healing (Clavien-Dindo grade IIIa), and one patient in whom CWPF was used to salvage implant had implant re-infection, necessitating implant removal (Clavien-Dindo grade IIIb). Details of the surgical procedures performed and outcomes are given in Table 2.

**Table 2:** Surgery performed – complications and outcomes CWPF: chest wall perforator flap; NSM: nipple-sparing mastectomy; LICAP: lateral intercostal artery perforator flap; MICAP: medial intercostal artery perforator flap; LTAP: lateral thoracic artery perforator flap; WLE: wide local excision; UIQ: upper inner quadrant; AICAP: anterior intercostal artery perforator flap; ANC: axillary node clearance; NAC: nipple-areola complex; SLNB: sentinel lymph node biopsy; DCIS: ductal carcinoma in situ.

Whole breast reconstruction							
	Procedure performed	Indication	Type of CWPF performed	Complications type	Clavien-Dindo grade	Follow-up (months)	Recurrence
1	Right NSM + reconstruction with chest wall perforator flaps + SLNB	Multi-focal tumour	LICAP + MICAP	Skin flap necrosis, delayed wound healing	IIIa	9	Nil
2	Left NSM+ recon with CWPF + SLNB	Recurrent tumour	LICAP + AICAP	Nil	NA	8	Nil
3	Right NSM + recon with CWPF + SLNB	Ca breast - persistent margin positivity	LICAP + MICAP	Nil	NA	12	Nil
4	Right NSM + recon with CWPF + SLNB	Multi-focal DCIS	LICAP + MICAP	Nil	NA	2	Nil
Salvage procedure for implant complications							
1	Right implant removal + capsulectomy + LICAP (with skin)	Implant extrusion on native skin	LICAP	Nil	NA	7	Nil
2	Left implant exchange + MICAP flap	Sinus and thin skin post-implant infection	MICAP	Implant re-infection	IIIb	3	Nil
Providing skin cover (for skin/NAC)							
1	Right breast wide local excision + LTAP flap (with skin) + SLNB	Large tumour close to the skin; skin paddle needed	LTAP (with skin)	Nil	NA	4	Nil
2	Left breast WLE + NAC + MICAP flap + left SLNB	Skin paddle for NAC reconstruction	MICAP (with skin)	Nil	NA	4	Nil
3	Left wide local excision (with skin) + sentinel lymph node biopsy + LICAP (with skin)	Large tumour close to the skin; skin paddle needed	LICAP (with skin)	Nil	NA	9	Nil
UIQ tumours							
1	Left breast WLE + AICAP flap + left ANC	UIQ tumour	AICAP	Nil	NA	17	Nil
2	Left breast WLE + MICAP flap	UIQ tumour	MICAP	Nil	NA	11	Nil

PROMs were measured for 10 patients. One patient could not be reached for the assessment. The PROMs assessment showed that overall all patients were satisfied with the post-operative breast appearance: 3/10 (30%) were very satisfied and 7/10 (70%) were satisfied with the result. Of the patients, 90% (9/10) felt the results of their surgery to be good, very good, or excellent.

Of the patients, 70% (7/10) said that they have no/little persistent pain. None of these patients had difficulty carrying out normal activities. Of the patients, 80% had no difficulty whatsoever in performing exercises or lifting heavy objects with the operated arm.

Overall, 60% (6/10) felt emotionally confident and feminine at least some of the time. Of the patients, 50% felt sexually attractive and comfortable most or all of the time, while the remaining half felt that their sexual life had been affected.

Details of PROMs assessed according to the type of procedure are shown in Table 3.

**Table 3:** Patient-reported outcome measures (PROMs) assessed at follow-up for patients undergoing CWPFs for extended indications CWPFs: chest wall perforator flaps; NAC: nipple-areola complex; UIQ: upper inner quadrant.

Whole breast reconstruction (n = 4)			
	Satisfied	Neutral	Dissatisfied
Satisfaction with surgery	4/4 (100%)	_	_
Satisfaction with the cosmetic outcome	4/4 (100%)	_	_
Physical well-being	3/4 (75%)	_	1/4 (25%)
Emotional well-being	2/3 (66.7%)	_	1/3 (33.3%)
Sexual well-being	2/3 (66.7%)		1/3 (33.3%)
Functional ability (carrying out normal activities)	4/4 (100%)	_	_
Salvage procedure for implant complications (n = 2)			
Satisfaction with surgery	2/2 (100%)	_	_
Satisfaction with the cosmetic outcome	2/2 (100%)	_	_
Physical well-being	2/2 (100%)	_	_
Emotional well-being	1/2 (50%)	1/2 (50%)	_
Sexual well-being	1/2 (50%)	1/2 (50%)	_
Functional ability (carrying out normal activities)	2/2 (100%)	_	_
Providing skin cover (for skin/NAC) (n = 2)			
Satisfaction with surgery	2/2 (100%)	_	_
Satisfaction with the cosmetic outcome	1/2 (50%)	1/2 (50%)	_
Physical well-being	2/2 (100%)	_	_
Emotional well-being	1/2 (50%)	_	1/2 (50%)
Sexual well-being	1/2 (50%)	_	1/2 (50%)
Functional ability (carrying out normal activities)	2/2 (100%)	_	_
UIQ tumours (n = 1)			
Satisfaction with surgery	1/1 (100%)	_	_
Satisfaction with the cosmetic outcome	1/1 (100%)	_	_
Physical well-being	1/1 (100%)	_	_
Emotional well-being	_	_	1/1 (100%)
Sexual well-being	_	_	1/1 (100%)
Functional ability (carrying out normal activities)	1/1 (100%)	_	_

## Discussion

The application of CWPFs to the above-mentioned scenarios is a novel application [14]. We have shown in our study that 81.81% of the patients underwent surgery without any complications, and all patients (100%) were either satisfied or very satisfied with the results of the surgery (as evaluated by the PROMs questionnaire). Also, there was minimal donor site morbidity, with none of the patients having any difficulty post-operatively in carrying out daily activities.

Tumours requiring skin cover/central quadrant tumours where NAC reconstruction is needed

Traditionally, these patients have been offered mastectomies. For central quadrant tumours in moderate to large beasts, Grisotti flap/inferior pedicle reduction mammoplasty can be applied but not in small non-ptotic breasts where volume replacement is needed [15,16]. Khallaf et al., in their study on different oncoplastic techniques for central quadrant tumours, reported a complication rate of 22.8% with an 88.6% patient satisfaction rate [16]. Nigam et al. have recently (2021) published a series of four patients, where they used CWPFs to reconstruct the NAC in central quadrant tumours [17]. They report no wound complications and very good-excellent aesthetic outcomes in all four patients.

This compares favourably with our study as three patients who underwent this surgery had no complications and all of them were satisfied with the results of the surgery.

Whole breast reconstruction

Implants as well as free/pedicled flaps described previously carry their own set of complications and resource limitations. Hence, we applied the use of double CWPFs for whole breast reconstruction in four patients. One of these patients had skin flap necrosis, which is a well-defined complication of nipple-sparing mastectomy, not related to the reconstructive technique per se. Also, the patient had a history of smoking 20 cigarettes per day, which is a well-documented risk factor for surgical complications [18]. All patients were satisfied with the cosmetic outcome. So far, any additional procedures or contralateral symmetrisation have not been done for any, although some patients may need lipo-grafting in the future. The use of CWPFs alone for whole breast reconstruction is a novel concept. There is an extreme paucity of data for this. Retrouvey et al. in their study from 2021 reported on six patients with mastectomy undergoing reconstruction with CWPFs [19]. But their study was heterogeneous, also including some patients where both expander and CWPF were used. Also, PROMs were not assessed, nor was any objective assessment of cosmetic outcomes done.

Nevertheless, we state that utmost discretion must be applied in selecting patients for whole breast reconstruction with CWPFs. This is a technique in the armamentarium but can be applied with expertise only to a very limited subset of patients who have large excess tissue on the lateral fold and upper abdomen but a small/medium breast. Contralateral symmetrisation or fat grafting of the ipsilateral breast will most likely be needed, especially after radiotherapy.

Upper inner quadrant tumours

While some UIQ tumours in moderate/large breasts can be tackled by certain oncoplastic techniques like batwing mastopexy and matrix rotation mammoplasty [2,20], most of these give sub-optimal aesthetic outcomes and are not adequate when the tumour/beast size ratio is not favourable. Hence, we present a relatively novel concept of using AICAP/MICAP flaps for providing cosmetically acceptable breast conservation surgery (BCS) even in this scenario. None of the patients had complications and the patients for whom PROMs could be done were satisfied with the cosmetic outcome.

Salvage procedure for implant complications

Two patients underwent LICAP and MICAP flaps as salvage procedures after implant extrusion on native skin. One had an implant exchange for implant extrusion on native skin with implant infection. Unfortunately, the patient had an implant re-infection, which necessitated the removal of the implant. However, because of the LICAP in situ, the patient had some breast volume, which may need further lipo-grafting.

The limitation of this study is that it is retrospective and includes a small number of patients with limited follow-up. And also, the study is heterogeneous; hence, the results have to be applied with discretion to broader populations. Also, other treatments like radiotherapy, chemotherapy, and hormonal therapy could have acted as confounding factors in the study, affecting the physical, emotional, and sexual well-being of the patient and hence, acting as sources of bias. Nevertheless, it does open up a number of existing options for a better solution to these problems.

## Conclusions

This is a novel study showing that the applications of CWPFs could be extended for whole breast reconstruction, management of implant complications, UIQ tumours, and for providing skin cover in a selected subset of patients with a low complication rate and a high patient satisfaction rate.

## Appendices

Patient-reported outcomes post chest wall perforator flap questionnaire (adapted from NMBRA study)

1. Patient responses on the items forming the “Satisfaction with information provision” scale. Each item could be answered as very dissatisfied, dissatisfied, satisfied, and very satisfied.

• How was the surgery to be done?

• Healing and recovery time?

• Possible complications?

• Options regarding types of partial breast reconstruction?

• Having a perforator flap versus regular breast conservation surgery?

• How long the procedure would take?

• What size you could expect your breasts to be?

• Any size discrepancy between the two breasts expected?

• How much pain to expect during recovery?

• What complications to expect?

• What you could expect your breasts to look like?

• How long it would take to feel like yourself/feel normal again?

• How the surgery would affect future breast cancer screening (e.g. mammograms)?

• Any alteration of sensation post-surgery in the breast or nipple?

• What do other women experience with their surgery?

• What the scars would look like?

2. Overall assessment of treatment after surgery:

• Overall, did you feel you were treated with respect and dignity while you were in the hospital?

Yes always, Yes, Sometimes, No

• Overall, how would you rate the care you received? Excellent, very good, good, fair, poor

3. Levels of satisfaction with consultant surgeon after surgery. Each item could be answered as very dissatisfied, dissatisfied, satisfied, very satisfied

• Was competent?

• Gave you confidence?

• Involved you in the decision-making process?

• Was reassuring?

• Answered all your questions?

• Made you feel comfortable?

• Was thorough?

• Was easy to talk to?

• Understood what you wanted?

• Was sensitive?

• Made time for your concerns?

• Was available when you had concerns?

4. Levels of satisfaction with the breast cancer clinical team (doctors and nurses) after surgery. Each item could be answered as very dissatisfied, dissatisfied, satisfied, very satisfied

• Were professional?

• Treated you with respect?

• Were knowledgeable?

• Were friendly and kind?

• Made you feel comfortable?

• Were thorough?

• Made time for your concerns?

5. Patients’ rating of the results of their surgery post-operatively

• Overall, how would you describe the results of your operation? Excellent, very good, good, fair, poor

Satisfaction with the postoperative appearance of the breast area after CWPF surgery. Each item could be answered as very dissatisfied, dissatisfied, satisfied, very satisfied

• How do you look in the mirror clothed?

• The shape of your operated breast when you are wearing a bra?

• How normal do you feel in your clothes?

• The size of your operated breast?

• Being able to wear clothing that is more fitted?

• How your breasts (unclothed) are lined up in relation to each other?

• How comfortably do your bras fit?

• The softness of your operated breast?

• How equal in size your breasts are to each other (unclothed)?

• How does your operated breast looks (unclothed)?

• How your operated breast feels to touch?

• How closely matched your breasts are to each other (unclothed)?

• How does your operated breast looks now compared to before you had any breast surgery?

• How do you look in the mirror unclothed?

6. Responses of women who underwent CWPF to the items within the scales for physical well-being, emotional well-being, and sexual well-being post-operatively

Patients could answer it as none of the time, little of the time, some of the time, most of the time, all of the time

Physical well-being

How often have you experienced:

• Neck pain?

• Upper back pain?

• Shoulder pain?

• Arm pain?

• Rib pain?

• Pain in the muscles in your chest?

• Difficulty lifting or moving your arms?

• Pain/tightness/pulling in the abdomen?

• Difficulty sleeping because of discomfort in your breast area?

• Tightness in your breast area?

• Pulling in your breast area?

• A nagging feeling in your breast area?

• Tenderness in your breast area?

• Sharp pains in your breast area?

• Shooting pains in your breast area?

• An aching feeling in your breast area?

• A throbbing feeling in your breast area?

** Swelling (lymphoedema) of the arm on the side where you had your axillary surgery?*

Emotional well-being

How often have you felt:

• Confident in a social setting?

• Emotionally able to do the things that you want to do?

• Emotionally healthy?

• Of equal worth to other women?

• Self-confident?

• Feminine in your clothes?

• Accepting your body?

• Normal? 

• Like other women?

• Attractive?

Sexual well-being

How often have you felt:

• Sexually attractive in your clothes?

• Comfortable/at ease during sexual activity?

• Confident sexually?

• Satisfied with your sex life?

• Confident sexually about how your breasts look when unclothed?

• Sexually attractive when unclothed?

7. Satisfaction with donor site scar (lateral scar) among women who had CWPFs

Patients could answer it as none of the time, little of the time, some of the time, most of the time, all of the time

• How your scar looks?

• The location of your scar?

• The length of your scar?

• How noticeable your scar is to others?

• Having to wear certain clothes in order to hide your scar?

• Not being able to wear certain clothes because of your scar (e.g. backless dress and bathing suit)?

8. Experience of functional impairment among women who had CWPFs

Patients could answer it as none of the time, little of the time, some of the time, most of the time, all of the time

For those undergoing LICAP flap:

• Shoulder pain?

• An aching feeling in your shoulder area?

• Shoulder stiffness?

• Tightness when you stretch your arm?

• Weakness in your arm?

• Difficulty in carrying out any of your normal activities?

• Difficulty in carrying out any exercise involving the operated arm?

• Difficulty carrying heavy objects (e.g. large bag of groceries)?

• Difficulty lifting heavy objects (e.g. large bag of groceries)?

• Difficulty reaching for objects (e.g. taking something down from a high shelf)?

• Difficulty doing activities with your arms outstretched (e.g. vacuuming and shovelling)?

• Difficulty doing activities with your arms above your head (e.g. doing up dress zipper and styling your hair)?

• Difficulty doing activities that repeatedly use shoulder & back muscles (e.g. throwing a ball, playing tennis, and swimming)?

For those undergoing AICAP flap:

• How often have you experienced:

• Abdominal discomfort?

• Tightness in your abdomen?

• Pulling in your abdomen?

• Difficulty doing everyday activities because of abdominal muscle weakness (e.g. making your bed)?
